# Sex-specific dysregulation of the *CX_3_CL1/CX_3_CR1* Axis following cocaine exposure: Translational evidence for a potential biomarker of abstinence

**DOI:** 10.1016/j.pnpbp.2025.111482

**Published:** 2025-08-31

**Authors:** Oscar Porras-Perales, Laura Sánchez-Marín, Dina Medina-Vera, María Flores-López, Laura Martín-Chaves, Milena Boccalon, Nerea Requena-Ocaña, Nuria García-Marchena, Raquel Reviriego, Luis J. Santín, Remi Martin-Fardon, Manuel Jiménez-Navarro, Fernando Rodríguez de Fonseca, Antonia Serrano, Francisco Javier Pavón-Morón

**Affiliations:** a Instituto de Investigación Biomédica de Málaga y Plataforma en Nanomedicina (IBIMA Plataforma BIONAND), Málaga, Spain; b Área del Corazón, Hospital Universitario Virgen de la Victoria, Málaga, Spain; c Facultad de Psicología, Universidad de Málaga, Málaga, Spain; d UGC Salud Mental, Hospital Regional Universitario de Málaga, Málaga, Spain; e Facultad de Medicina, Universidad de Málaga, Málaga, Spain; f CIBER de Enfermedades Cardiovasculares (CIBERCV), Instituto de Salud Carlos III, Madrid, Spain; g Facultad de Psicología, Universidad Complutense de Madrid, Madrid, Spain; h Department of Translational Medicine, Scripps Research, La Jolla, CA, USA; i Unidad Clínica de Neurología, Hospital Regional Universitario de Málaga, Málaga, Spain

**Keywords:** Abstinence, Biomarker, Cocaine, Fractalkine, Sexual dimorphism, Translational

## Abstract

Cocaine disrupts neurotransmitter systems and promotes neuroinflammation by activating microglia and altering cytokine signaling. The CX_3_CL1/CX_3_CR1 axis is an essential signaling pathway for microglial regulation, may exhibit sex-specific responses to cocaine. In this study, male and female Wistar rats were exposed to acute (5, 15, or 30 mg/kg) or repeated (15 mg/kg/day for two weeks) cocaine. Gene expression of *Cx3cl1* and *Cx3cr1* was assessed in the amygdala and hippocampus, alongside plasma CX_3_CL1 concentrations. Additionally, plasma CX_3_CL1 concentrations were assessed in 88 abstinent patients with cocaine use disorder (CUD) and 30 matched healthy controls. Female rats exhibited significantly lower baseline mRNA expression of *Cx3cl1* and *Cx3cr1* in both brain regions compared with male rats. Acute cocaine induced dose- and time-dependent transcriptional changes, with female rats exhibiting more pronounced and sustained *Cx3cl1* and *Cx3cr1* expression changes compared with males. Repeated exposure produced sex-, region-, and abstinence-dependent regulations of *Cx3cl1* and *Cx3cr1*, with persistent downregulation of *Cx3cl1* and compensatory *Cx3cr1* upregulation in female rats. In plasma, only male rats exhibited elevated CX_3_CL1 concentrations following cocaine exposure, particularly during early abstinence (*i.e.*, 2 h–72 h). In humans, overall CX_3_CL1 concentrations did not differ between CUD patients and controls. However, CX_3_CL1 concentrations increased with abstinence duration, particularly in males (*r* = +0.34, *p* < 0.01), but not in females. These findings highlight sex-specific regulation of the CX_3_CL1/CX_3_CR1 axis in cocaine-induced neuroinflammation and suggest that plasma CX_3_CL1 concentrations may serve as a potential biomarker or contribute to a composite biosignature, together with other biomolecules, of CUD progression and abstinence. Considering sex differences, may enhance our understanding of addiction pathophysiology and inform targeted therapeutic strategies.

## 1. Introduction

Cocaine is a potent psychostimulant that disrupts brain function by interfering with key neurotransmitter systems, primarily enhancing monoamine signaling through the blockade of dopamine, norepinephrine, and serotonin transporters. Its main action occurs in the dopaminergic mesocorticolimbic pathway, a critical component of the brain’s reward system (Howell and Kimmel, 2008). Cocaine also triggers neuroinflammation, activating microglia and releasing pro-inflammatory cytokines, thereby disrupting emotional regulation, stress responses, and cognitive functions (Clark et al., 2013; Coller and Hutchinson, 2012). Furthermore, cocaine impairs neuroplasticity by reducing dendritic spine density and affecting synaptic connections, thus reinforces addictive behaviors (Robinson and Kolb, 2004).

CX3-C motif chemokine ligand 1 (CX_3_CL1), also known as fractalkine, is a distinctive chemokine found in both soluble and membrane-bound forms (Bazan et al., 1997). It binds to its receptor CX_3_CR1, which is predominantly expressed on microglia, playing a key role in regulating microglial activity and maintaining neuronal health (Paolicelli et al., 2014; Sheridan and Murphy, 2013). However, cocaine exposure disrupts this signaling pathway, leading to excessive microglial activation and exacerbated neuroinflammation (Montesinos et al., 2020). Alterations in the CX_3_CL1/CX_3_CR1 axis contribute to neural dysfunction in brain regions associated with reward and stress, exacerbating addiction pathology.

Given the potential for peripheral inflammatory changes to reflect neuroinflammation, CX_3_CL1 and CX_3_CR1 have been proposed as potential biomarkers for cocaine use disorder (CUD), a significant public health concern (Kampman, 2019). We previously reported an association between plasma CX_3_CL1 concentrations and addiction severity in abstinent patients with CUD, with higher levels observed in those with more severe symptoms (Araos et al., 2015). These circulating concentrations are also altered in mice after both acute and chronic cocaine exposure (Araos et al., 2015).

Significant evidence indicates sex differences in the prevalence and progression of CUD (Kennedy et al., 2013; Quigley et al., 2021). While men are more likely to initiate cocaine use, women tend to progress more rapidly to addiction and experience more intense cravings and stress-induced relapse (Quinones-Jenab and Jenab, 2012; Robbins et al., 1999; Winhusen and Lewis, 2013). Sex-specific patterns in the CX_3_CL1/CX_3_CR1 signaling pathway, driven by neuroinflammatory mechanisms, may help explain these differences. Notably, a recent study demonstrated sex-based variations in CX_3_CL1 modulation in rats following exposure to allopregnanolone, a neurosteroid with anti-inflammatory properties (Balan et al., 2024). Furthermore, CX_3_CR1-deficient mice exhibit maladaptive coping behaviors and heightened neuroinflammatory responses following adolescent stress and alcohol exposure, highlighting the critical role of this receptor in modulating stress-related neuroimmune pathways and vulnerability to substance use disorders (Medina-Vera et al., 2025). As cocaine influences neuroinflammation and CX_3_CL1 levels, these findings suggest that females may exhibit a distinct neuroinflammatory response compared with males.

We specifically focused on the amygdala and hippocampus because these limbic structures are key integrators of emotional regulation, associative and contextual memory, and stress responsivity, functions critically engaged during cocaine exposure, withdrawal, and relapse. In humans, high-resolution PET studies using [^18^F]fallypride have demonstrated that cocaine-related cues induce significant dopamine release in both the amygdala and hippocampus, supporting their role in emotional processing and relapse-related behaviors (Fotros et al., 2013). The dorsal hippocampus has been shown to mediate relapse behavior following prolonged cocaine abstinence in preclinical models, highlighting its involvement in relapse mechanisms (Werner et al., 2020). Moreover, both the amygdala and hippocampus exhibit stress-induced microglial remodeling and cytokine regulation, reinforcing their role in neuroimmune modulation under conditions relevant to addiction relapse (Nowak et al., 2025).

We hypothesize that sex differences in CX_3_CL1 regulation and astrocyte activity may underlie vulnerabilities to the effects of cocaine, influencing addiction progression, relapse rates, and treatment outcomes. To investigate this, we assessed *Cx3cl1* and *Cx3cr1* gene expression in the amygdala and hippocampus of male and female Wistar rats exposed to cocaine, evaluating different acute doses and the effects of abstinence duration following repeated cocaine exposure. Plasma CX_3_CL1 concentrations were also measured in these animals. Additionally, we extended our research to patients with CUD, examining the impact of cocaine use on the CX_3_CL1/CX_3_CR1 signaling pathway, particularly in relation to abstinence duration.

## 2. Materials / subjects and methods

### 2.1. Animals and ethics statements

Wistar rats of both sexes (Charles River Laboratories España S.A., Barcelona, Spain), weighing 200–250 g at the onset of the study, were housed in the Animal Resource Center at the University of Málaga (Spain). The animals were maintained under a 12-h light-dark cycle and kept in a room with controlled humidity and temperature throughout the experiments. All procedures adhered to the European Directive 2010/63/EU on the protection of animals used for scientific purposes and complied with Spanish regulations on laboratory animal care and use (RD 53/2013 and 178/2004, Ley 32/2007 and 9/2003, and Decreto 320/2010). Efforts were made to reduce the number of animals used and minimize pain or distress. All protocols were approved by the Ethic and Research Committee of the Universidad de Málaga (CEUMA) (#ID procedure: 2023–0002).

### 2.2. Cocaine treatments for rats

Cocaine (Merck Life Science, Madrid, Spain) was dissolved in sterile saline (0.9 % NaCl). Cocaine or vehicle (saline) was administered *via* intraperitoneal (i.p.) injection at a volume of 1 mL/kg body weight.

#### 2.2.1. Acute cocaine treatment

For acute treatment, male and female rats received cocaine at doses of 5, 15, or 30 mg/kg. The animals were euthanized 30 or 240 min after administration of cocaine or vehicle. Rats were randomly assigned to either the cocaine or vehicle groups (*N* = 6–7 rats of each sex per group/time point).

#### 2.2.2. Repeated cocaine treatment

For repeated treatment, male and female rats were administered cocaine at a dosage of 15 mg/kg (i.p.) daily for 2 weeks. Following the final injection, rats were euthanized at 2, 72, or 240 h. Control rats received vehicle injections under the same conditions as the cocaine-treated groups. Each group consisted of 6–8 animals of each sex.

### 2.3. Blood and brain sample collection from rats

Following cocaine or vehicle treatments, rats were anesthetized with sodium pentobarbital (50 mg/kg, i.p.) and blood and brain samples were collected.

#### 2.3.1. Plasma samples

Specifically, trunk blood samples were collected following guillotine decapitation using 6-mL BD Vacutainer^™^ Plastic Blood Collection Tubes containing K_2_EDTA (Becton, Dickinson and Company, Franklin Lakes, NJ, USA). The samples were immediately centrifuged at 2000 ×*g* for 15 min at 4 °C, and plasma aliquots obtained from the supernatant were stored at −80 °C until analysis.

#### 2.3.2. Dissection coordinates and tissue sampling

After rapid extraction, brains were frozen on dry ice, stored at −80 °C, and placed in an acrylic rat brain matrix. Coronal slabs of 2 mm thickness were generated from anterior to posterior. Guided by the adult rat brain atlas of Paxinos and Watson (Büttner-Ennever, 1997)., sampling targeted the following ranges (all coordinates relative to bregma): (1) Amygdala (bilateral; basolateral/central complex): AP ≈ −2.0 to −3.6 mm, ML ≈ 3.5–5.5 mm, DV ≈ 6.5–8.5 mm (from the dorsal brain surface in coronal slabs). (2) Dorsal hippocampus (bilateral; CA1/CA3/dentate gyrus): AP ≈ −2.8 to −5.2 mm, ML ≈ 1.5–4.0 mm, DV ≈ 2.0–4.0 mm.

Within these slabs, tissue was collected bilaterally using a sterile cylindrical sample corer (1.5–2.0 mm); punches were centered on the target nuclei/fields avoiding ventricles and white matter tracts. For each region, two punches per hemisphere were pooled to increase yield while maintaining regional specificity. Samples were immediately transferred to pre-chilled tubes on dry ice and stored at −80 °C until RNA extraction. All dissections were performed by trained personnel blinded to treatment/sex.

### 2.4. Determination of CX_3_CL1 concentrations in the plasma of rats

Soluble CX_3_CL1 concentrations in plasma samples were measured using a commercially available enzyme-linked immunoassay (ELISA) [Fractalkine (CX3CL1) Rat ELISA Kit] (catalog # ab100760, Abcam, Cambridge, UK) according to the manufacturer’s instructions. The ELISA was performed using 100 μL of plasma (3-fold dilution). Raw data, expressed as mean absorbance, were obtained immediately after adding the stop solution to each well at 450 nm. Plasma CX_3_CL1 concentrations were reported in pg/mL. The assay sensitivity was <5 pg/mL, with a standard curve range of 2.74 to 2000 pg/mL. Coefficients of variation for inter-assay and intra-assay were 9.7 % and 7.2 %, respectively.

### 2.5. RNA isolation and RT-qPCR analysis in the brain of rats

Real-time PCR was employed to quantify the relative mRNA levels of *Cx3cl1* (which encodes CX_3_CL1) and *Cx3cr1* (CX_3_CR1). Total RNA was extracted from brain samples using TRIzol^™^ Reagent according to the manufacturer’s instruction (Invitrogen, Thermo Fisher Scientific, Waltham, MA, USA), which includes tissue homogenization, phase separation with chloroform, RNA precipitation with isopropanol, ethanol washing, and resuspension in RNase-free water. RNA concentrations were measured spectrophotometrically, ensuring an absorbance ratio of 260/280 nm between 1.8 and 2.0. Reverse transcription was performed with the Transcriptor Reverse Transcriptase kit and random hexamer primers (Roche Diagnostic, Mannheim, Germany). RT-qPCR was conducted on an ABI PRISM 7300 Real-Time PCR System (Applied Biosystems, Foster City, CA, USA) using TaqMan Gene Expression Assays (Thermo Fisher Scientific, Waltham, MA, USA) with the FAM dye label format. All values were normalized to the housekeeping gene β-actin (*Actb*), which was stable across subgroups in the amygdala and hippocampus. Relative quantification was calculated using the ΔΔCt method, with results expressed as fold change relative to the corresponding control group [*e.g.*, male, dose 0 (vehicle); dose 0 (vehicle); male, vehicle at 2 h; and vehicle 2 h]. Accordingly, the y-axis in the figures is labeled as relative mRNA expression (fold change *vs.* control). Primer sequences were obtained from the TaqMan Assays genome database for rat mRNA analysis [Rn] (Table S1).

### 2.6. Patients with cocaine use disorder

This translational research included an exploratory study involving patients diagnosed with lifetime CUD who were in abstinence. Over a three-year period (March 2020–January 2023), 88 Caucasian individuals (69 men and 19 women) were recruited from outpatient cocaine treatment programs at the Centro Provincial de Drogodependencias (Málaga, Spain). Additionally, 30 healthy participants, matched for sex, BMI, and age, were recruited as a control group.

#### 2.6.1. Eligibility criteria

Participants met the following inclusion criteria: written informed consent, aged 18 to 65 years, a diagnosis with lifetime CUD, abstinence duration of less than one year, no recent chronic inflammatory or infectious diseases, no history of cancer, no cognitive or language limitations, and for women, not pregnant or breastfeeding. Although additional lifetime substance use disorders were permitted, cocaine had to be the primary substance for which treatment was sought.

#### 2.6.2. Ethical statement

Written informed consent was obtained from each participant after receiving a detailed explanation of the study. The study protocols and procedures were approved by the Ethics Committee of the Andalusian Health Service (Portal de Ética de la Investigación Biomédica de Andalucía-PEIBA, Consejería de Salud y Familias, Junta de Andalucía) (# code: 0813-N-23 // PI22/01833). This approval adhered to the Ethical Principles for Medical Research Involving Human Subjects outlined in the Declaration of Helsinki (64th WMA General Assembly, Fortaleza, Brazil, October 2013), Recommendation No. R (97) 5 on the Protection of Medical Data (1997), and the Spanish Data Protection Act [Regulation (EU) 2016/679 of the European Parliament and of the Council of 27 April 2016 on the protection of individuals with regard to personal data processing]. All collected data were coded to ensure privacy and confidentiality.

### 2.7. Clinical assessments

All participants were assessed by trained and experienced psychologists using diagnostic interviews. Substance use disorders and other mental disorders were diagnosed according to the DSM criteria, utilizing the Spanish version of the Psychiatric Research Interview for Substance and Mental Disorders (PRISM) (Torrens et al., 2004). PRISM is a semi-structured interview with well-established psychometric properties for the assessment of substance use disorders and major psychiatric comorbidities in populations with substance dependence (Hasin, 2015; Hasin et al., 1996).

### 2.8. Blood collection and processing

Blood extractions were conducted under standardized conditions by experienced nurses in the morning, following an 8–12-h fasting period. Venous blood was drawn into 9-mL K2 EDTA tubes (BD, Franklin Lakes, NJ, USA) and immediately processed to obtain plasma. Plasma was separated by centrifuging blood samples at 2200 ×*g* for 15 min at 4 °*C. prior* to centrifugation, rapid antigen detection tests for HIV, hepatitis B, hepatitis C, and COVID-19 were conducted on all samples. Samples that tested positive were handled and excluded following laboratory safety protocols. Plasma samples were logged and stored at −80 °C until further analysis.

### 2.9. Determination of CX_3_CL1 concentrations in the plasma of humans

Plasma CX_3_CL1 concentrations were measured using a human ProcartaPlex^™^ Simplex Kit Invitrogen^™^ (catalog # EPX01A-12,121–901, Thermo Fisher Scientific, Waltham, MA, USA) on a Luminex 200 Instrument System (Thermo Fisher Scientific, Waltham, MA, USA) at the Proteomics Unit of the Central Research Support Services, University of Malaga. The assay was performed using 25 μL of plasma, following the manufacturer’s instructions. Raw data, expressed as mean fluorescence intensity, were obtained after a 60-min plate reading. Plasma CX_3_CL1 concentrations were reported in pg/mL. The assay sensitivity was 20.5 pg/mL, with a standard curve range of 2.08 to 8500 pg/mL. Coefficients of variation for inter-assay and intra-assay were 7.2 % and 6.4 %, respectively.

### 2.10. Statistical analysis

All animal data are presented as the mean ± standard error of the mean (SEM). First, baseline levels of genes (*Cx3cl1* and *Cx3cr1*) in the amygdala and hippocampus, as well as baseline CX_3_CL1 concentrations, were compared between male and female control groups using Student’s *t*-test to assess sex differences. Next, to evaluate the dose–response effects of acute cocaine on genes and protein concentrations, a one-way analysis of variance (ANOVA) was performed separately for each sex, followed by Tukey’s test for *post hoc* multiple comparisons. In the repeated-treatment experiments, data were analyzed by two-way ANOVA for each sex [factor 1 (f1) = “cocaine” (levels: vehicle and cocaine); factor 2 (f2) = “time of abstinence” (levels: 2 h, 72 h, and 240 h)]. To examine sex differences in the vehicle groups over time, a two-way ANOVA was also conducted [factor 1 (f1) = “sex” (levels: male and female); factor 2 (f2) = “time of abstinence” (levels: 2 h, 72 h, and 240 h)]. Sidak’s test was used for *post hoc* pairwise comparisons when significant interactions (f1 × f2) were detected.

For the human study, plasma CX_3_CL1 concentrations [mean ± standard deviation (SD)] at different durations of cocaine abstinence were assessed by one-way ANOVA, followed by Tukey’s test for *post hoc* comparisons. Pearson correlation analyses were then performed between plasma CX_3_CL1 concentrations and abstinence duration (days) after log_10_-transformation of the abstinence period.

Test statistics and degrees of freedom are reported in the Results where appropriate. A *p* < 0.05 was considered statistically significant. All statistical analyses were performed using GraphPad Prism version 5.04 (GraphPad Software, San Diego, CA).

## 3. Results

### 3.1. Effects of cocaine exposure on the mRNA expression of Cx3cl1 and Cx3cr1 in the amygdala of rats

#### 3.1.1. Acute cocaine treatment

##### 3.1.1.1. Cx3cl1 at 30 min.

**Fig. 1.**
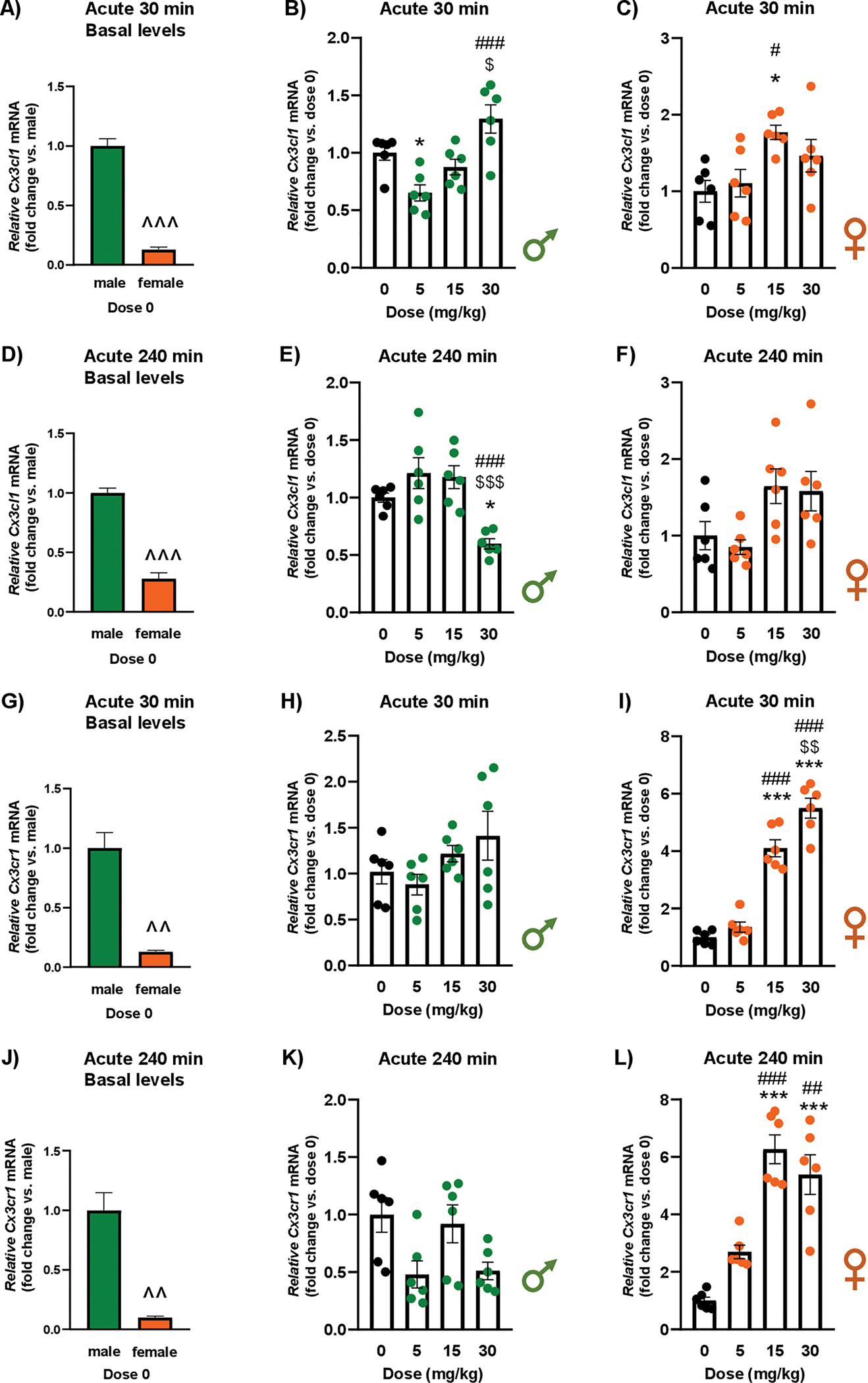
Effects of acute cocaine exposure on the relative mRNA expression of *Cx3cl1* and *Cx3cr1* in the amygdala of rats. Basal *Cx3cl1* mRNA levels in male and female control rats 30 min after vehicle administration (fold change vs. males, dose 0) **(A)**. Relative *Cx3cl1* mRNA levels in male **(B)** and female rats **(C)** 30 min after vehicle or cocaine treatment. Basal *Cx3cl1* mRNA levels in male and female control rats 240 min after vehicle administration (fold change vs. males, dose 0) **(D)**. Relative *Cx3cl1* mRNA levels in male **(E)** and female rats **(F)** 240 min after vehicle or cocaine treatment. Basal *Cx3cr1* mRNA levels in male and female control rats 30 min after vehicle administration (fold change vs. males, dose 0) **(G)**. Relative *Cx3cr1* mRNA levels in male **(H)** and female rats **(I)** 30 min after vehicle or cocaine treatment. Basal *Cx3cr1* mRNA levels in male and female control rats 240 min after vehicle administration (fold change vs. males, dose 0) **(J)**. Relative *Cx3cr1* mRNA levels in male **(K)** and female rats **(L)** 240 min after vehicle or cocaine treatment. Basal mRNA levels were analyzed using Student’s *t*-test. Relative *Cx3cl1* and *Cx3cr1* mRNA levels were analyzed by one-way ANOVA in male and female rats. Bars represent the mean ± SEM (6–7 rats/group). (^^) *p* < 0.01 and (^^^) *p* < 0.001 indicate significant differences compared with control males/dose 0. (*) *p* < 0.05 and (***) *p* < 0.001 indicate significant differences compared with the vehicle group. (#) *p* < 0.05, (##) *p* < 0.01, and (###) *p* < 0.001 indicate significant differences compared with the group treated with 5 mg/kg. ($) *p* < 0.05, ($ $) *p* < 0.01, and ($ $ $) *p* < 0.001 indicate significant differences compared with the group treated with 15 mg/kg.

The analysis revealed significant differences in basal *Cx3cl1* mRNA expression between male and female rats (t_6_ = 13.76; *p* < 0.001), with females exhibiting significantly lower relative mRNA levels than males under control conditions (*i.e.*, vehicle or dose 0) (Fig. 1A).

In male rats, a one-way ANOVA revealed a significant main effect of “dose of cocaine” on *Cx3cl1* mRNA expression of (F_3,20_ = 9.94; *p* < 0.001) at 30 min after treatment (Fig. 1B). *Post hoc* tests for multiple comparisons showed that male rats treated with 5 mg/kg displayed lower relative *Cx3cl1* mRNA levels than vehicle-treated rats (*p* < 0.05). Additionally, male rats treated with the higher dose of cocaine displayed higher relative *Cx3cl1* mRNA levels compared with those treated with 5 mg/kg (*p* < 0.001) and 15 mg/kg (*p* < 0.05). In female rats, there was also a significant main effect of “dose of cocaine” at 30 min (F_3,20_ = 4.63; *p* = 0.013) (Fig. 1C). *Post hoc* comparisons indicated that females treated with 15 mg/kg exhibited higher relative *Cx3cl1* mRNA levels than both vehicle-treated rats (*p* < 0.05) and those treated with 5 mg/kg (*p* < 0.05).

##### 3.1.1.2. Cx3cl1 at 240 min.

Significant differences were observed in basal *Cx3cl1* mRNA expression between male and female vehicle-treated rats (t_10_ = 11.24; *p* < 0.001), with female rats showing significantly lower relative mRNA levels than males under control conditions (Fig. 1D).

In male rats, there was a significant main effect of “dose of cocaine” on *Cx3cl1* mRNA expression at 240 min (F_3,20_ = 10.12; *p* < 0.001) (Fig. 1E). *Post hoc* comparisons showed that males treated with 30 mg/kg had lower relative mRNA levels than those treated with vehicle (*p* < 0.05), 5 mg/kg (*p* < 0.001), and 15 mg/kg (*p* < 0.001). In female rats, there was a significant main effect of “dose of cocaine” on *Cx3cl1* mRNA expression at 240 min (F_3,20_ = 4.03; *p* = 0.022) (Fig. 1F). Although higher cocaine doses increased relative *Cx3cl1* mRNA levels, this effect did not reach statistical significance.

##### 3.1.1.3. Cx3cr1 at 30 min.

There were significant differences in basal *Cx3cr1* mRNA expression between male and female vehicle-treated rats (t_5_ = 6.67; *p* = 0.001), with females exhibiting significantly lower relative mRNA levels compared with males (Fig. 1G).

In male rats, no significant differences were observed at 30 min after the cocaine treatment (Fig. 1H). In contrast, female rats showed a significant main effect of “dose of cocaine” on *Cx3cr1* expression (F_3,20_ = 76.82; *p* < 0.001) (Fig. 1I). *Post hoc* comparisons indicated that females treated with 15 and 30 mg/kg cocaine had higher relative mRNA levels than both vehicle-treated rats (*p* < 0.001) and those treated with 5 mg/kg (*p* < 0.001). Additionally, the highest dose of cocaine produced significantly higher *Cx3cr1* mRNA levels than 15 mg/kg (*p* < 0.01).

##### 3.1.1.4. Cx3cr1 at 240 min.

The analysis revealed significant differences in basal *Cx3cr1* expression between male and female rats under control conditions (t_5_ = 5.99; *p* = 0.002), with females exhibiting significantly lower relative mRNA levels compared with vehicle-treated male rats (Fig. 1J).

In male rats, a two-way ANOVA indicated a significant main effect of “dose of cocaine” on *Cx3cr1* expression at 240 min (F_3,20_ = 4.11; *p* = 0.020), but *post hoc* tests did not detect statistically significant pairwise differences, despite a trend toward decreased relative *Cx3cr1* mRNA in cocaine-treated males (Fig. 1K). In female rats, there was a significant main effect of “dose of cocaine” at 240 min (F_3,20_ = 29.45; *p* < 0.001) (Fig. 1L). *Post hoc* tests showed that females treated with 15 and 30 mg/kg of cocaine had higher relative *Cx3cr1* mRNA levels than both vehicle-treated rats (*p* < 0.001) and those treated with 5 mg/kg (*p* < 0.001).

### 3.1.2. Repeated cocaine treatment

#### 3.1.2.1. Cx3cl1.

**Fig. 2.**
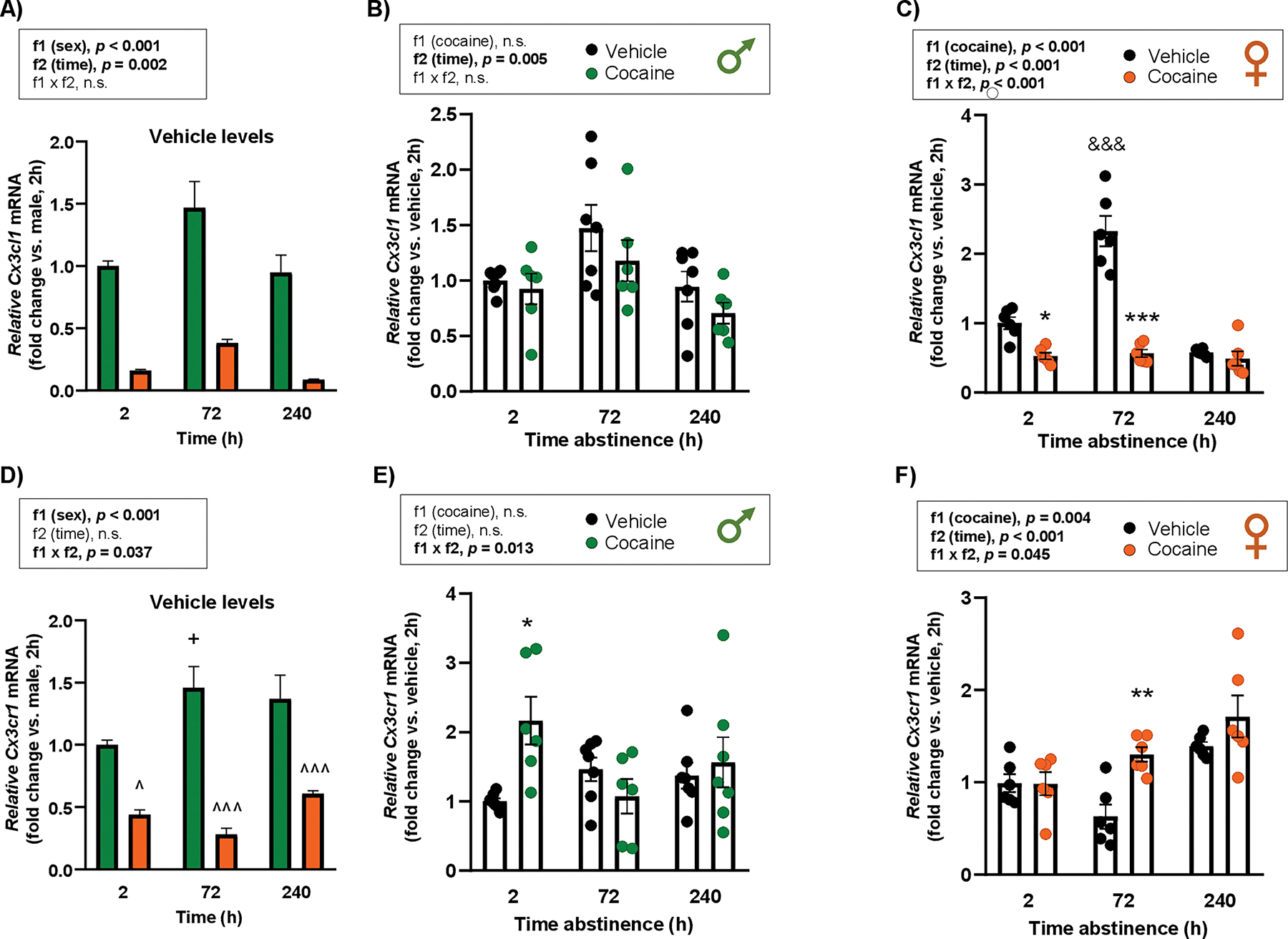
Effects of repeated cocaine exposure on the relative mRNA expression of *Cx3cl1* and *Cx3cr1* in the amygdala of rats. Basal *Cx3cl1* mRNA levels in male and female control rats at different time points after vehicle administration (fold change vs. males, 2 h) **(A)**. Relative *Cx3cl1* mRNA levels in male **(B)** and female rats **(C)** 2, 72, and 240 h after vehicle or cocaine treatment. Basal *Cx3cr1* mRNA levels in male and female control rats at different time points after vehicle administration (fold change vs. males, 2 h) **(D)**. Relative *Cx3cr1* mRNA levels in male **(E)** and female rats **(F)** 2, 72, and 240 h after vehicle or cocaine treatment. Relative mRNA levels were analyzed by two-way ANOVA. Bars represent the mean ± SEM (6–8 rats/group). (^) *p* < 0.05 and (^^^) *p* < 0.001 indicate significant differences compared with males. (+) *p* < 0.05 indicates significant differences compared with control males at 2 h. (*) *p* < 0.05, (**) *p* < 0.01, and (***) *p* < 0.001 indicate significant differences compared with their respective vehicle group. (&&&) *p* < 0.001 indicates significant differences compared with the vehicle group at 2 h.

First, we compared vehicle groups between males and females. A two-way ANOVA revealed significant main effects of “sex” (F1,33 = 99.77; *p* < 0.001) and “time” (F2,33 = 7.35; *p* = 0.002) (Fig. 2A). Male rats treated with vehicle had higher relative *Cx3cl1* mRNA levels in the amygdala than females at all time points.

In male rats, there was a significant main effect of “time of abstinence” (F_2,33_ = 6.28; *p* = 0.005) on *Cx3cl1* mRNA expression, with no main effect of cocaine exposure or interaction (Fig. 2B). In female rats, a two-way ANOVA revealed significant main effects of “cocaine” (F_1,30_ = 75.31; *p* < 0.001) and “time of abstinence” (F_2,30_ = 37.51; *p* < 0.001), as well as a significant interaction (F_2,30_ = 32.01; *p* < 0.001) (Fig. 2C). *Post hoc* comparisons showed a significant reduction in relative *Cx3cl1* mRNA levels in cocaine-treated females at 2 h (*p* < 0.05) and 72 h (*p* < 0.001) of abstinence compared with their respective vehicle rats. Moreover, female vehicle-treated rats displayed higher relative mRNA levels at 72 h than at 2 h (*p* < 0.001).

##### 3.1.2.2. Cx3cr1.

When comparing *Cx3cr1* mRNA expression in males and females treated with vehicle, there was a significant main effect of “sex” (F_1,33_ = 75.75; *p* < 0.001) and a significant interaction between “sex” and “time” (F_2,33_ = 3.64; *p* = 0.037) (Fig. 2D). *Post hoc* tests revealed that female rats had lower relative *Cx3cr1* mRNA levels than males at all time points (2 h: *p* < 0.05; 72 h and 240 h: *p* < 0.001) and that male rats at 72 h exhibited higher *Cx3cr1* mRNA compared with males at 2 h (*p* < 0.05).

In male rats, there was a significant interaction between “cocaine” and “time of abstinence” (F_2,34_ = 4.96; *p* = 0.013) (Fig. 2E). *Post hoc* comparisons showed that cocaine-treated male rats had higher relative *Cx3cr1* mRNA levels than vehicle-treated rats (*p* < 0.05) at 2 h of abstinence. In female rats, a two-way ANOVA revealed significant main effects of “cocaine” (F_1,30_ = 9.61; *p* = 0.004) and “time of abstinence” (F_2,30_ = 13.10; *p* < 0.001), as well as a significant interaction (F_2,30_ = 3.42; *p* = 0.045) (Fig. 2F). *Post hoc* tests indicated a significant increase in relative *Cx3cr1* mRNA in cocaine-treated females (*p* < 0.01) at 72 h of abstinence compared with their vehicle-treated rats.

### 3.2. Effects of cocaine exposure on the mRNA expression of Cx3cl1 and Cx3cr1 in the Hippocampus of rats

#### 3.2.1. Acute cocaine treatment

##### 3.2.1.1. Cx3cl1 at 30 min.

**Fig. 3.**
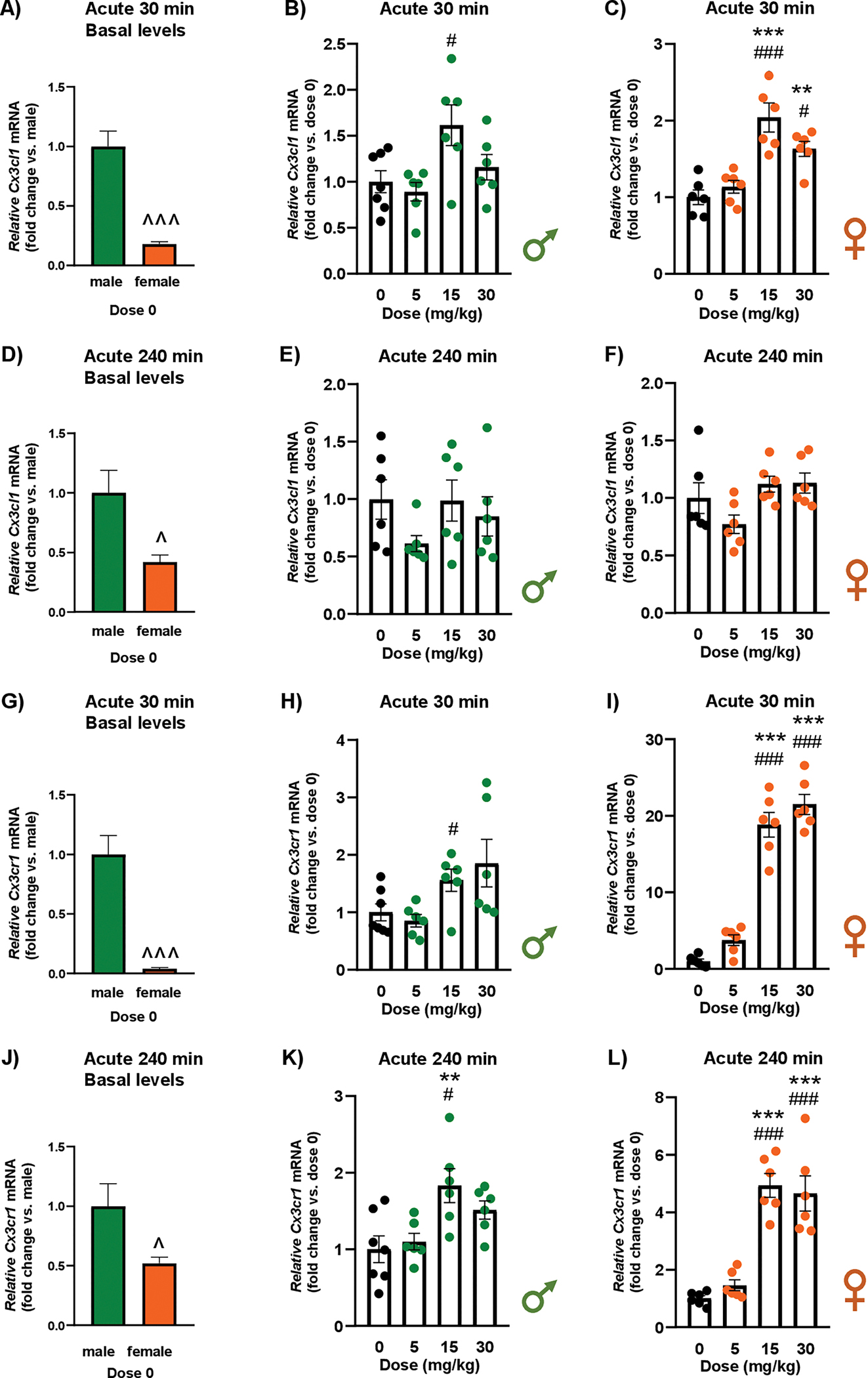
Effects of acute cocaine exposure on the relative mRNA expression of *Cx3cl1* and *Cx3cr1* in the hippocampus of rats. Basal *Cx3cl1* mRNA levels in male and female control rats 30 min after vehicle administration (fold change vs. males, dose 0) **(A)**. Relative *Cx3cl1* mRNA levels in male **(B)** and female rats **(C)** 30 min after vehicle or cocaine treatment. Basal *Cx3cl1* mRNA levels in male and female control rats 240 min after vehicle administration (fold change vs. males, dose 0) **(D)**. Relative *Cx3cl1* mRNA levels in male **(E)** and female rats **(F)** 240 min after vehicle or cocaine treatment. Basal *Cx3cr1* mRNA levels in male and female control rats 30 min after vehicle administration (fold change vs. males, dose 0) **(G)**. Relative *Cx3cr1* mRNA levels in male **(H)** and female rats **(I)** 30 min after vehicle or cocaine treatment. Basal *Cx3cr1* mRNA levels in male and female control rats 240 min after vehicle administration (fold change vs. males, dose 0) **(J)**. Relative *Cx3cr1* mRNA levels in male **(K)** and female rats **(L)** 240 min after vehicle or cocaine treatment. Basal mRNA levels were analyzed using Student’s *t*-test (Fig. 3A, 3D, 3G, and 3J). Relative mRNA levels were analyzed by one-way ANOVA in male and female rats. Bars represent the mean ± SEM (6–7 rats/group). (^) *p* < 0.05 and (^^^) *p* < 0.001 indicate significant differences compared with control males/dose 0. (*) *p* < 0.05, (**) *p* < 0.01, and (***) *p* < 0.001 indicate significant differences compared with the vehicle group. (#) *p* < 0.05 and (###) *p* < 0.001 indicates significant differences compared with the group treated with 5 mg/kg.

The analysis revealed significant differences in basal *Cx3cl1* mRNA levels between male and female rats under control conditions (*i.e.*, vehicle or dose 0) (t_6_ = 6.23; *p* < 0.001), with females showing significantly lower relative mRNA levels compared with males (Fig. 3A).

In male rats, there was a significant main effect of “dose of cocaine” on relative *Cx3cl1* mRNA expression at 30 min (F_3,21_ = 4.39; *p* = 0.016) (Fig. 3B). *Post hoc* tests showed that males treated with 15 mg/kg had higher relative mRNA levels than vehicle-treated rats (*p* < 0.05) and those treated with 5 mg/kg (*p* < 0.05). In female rats, a significant main effect of “dose of cocaine” was observed on *Cx3cl1* mRNA expression at 30 min (F_3,20_ = 14.86; *p* < 0.001) (Fig. 3C). Females treated with 15 and 30 mg/kg had higher relative mRNA levels than vehicle-treated rats (*p* < 0.001 and *p* < 0.01, respectively) and those treated with 5 mg/kg (*p* < 0.001 and *p* < 0.05, respectively).

##### 3.2.1.2. Cx3cl1 at 240 min.

Significant differences were observed in relative *Cx3cl1* mRNA levels between male and female vehicle-treated rats (t_6_ = 2.91; *p* = 0.027), with females showing significantly lower relative mRNA levels than male rats (Fig. 3D).

However, no significant effect of “dose of cocaine” on *Cx3cl1* mRNA expression was detected in either males (Fig. 3E) or females (Fig. 3F) at 240 min after treatment.

##### 3.2.1.3. Cx3cr1 at 30 min.

When comparing *Cx3cr1* mRNA expression in male and female vehicle-treated rats at 30 min, there were significant differences (t_6_ = 5.99; *p* < 0.001) and females exhibited significantly lower relative mRNA levels than males (Fig. 3G).

In male rats, there was a significant main effect of “dose of cocaine” at 30 min (F_3,21_ = 3.79; *p* = 0.026) (Fig. 3H). *Post hoc* tests showed that males treated with 15 mg/kg exhibited higher relative *Cx3cr1* mRNA levels than those treated with 5 mg/kg (*p* < 0.05). In female rats, there was a significant main effect of “dose of cocaine” at 30 min (F_3,20_ = 87.87; *p* < 0.001) (Fig. 3I). *Post hoc* tests showed that females treated with 15 and 30 mg/kg had higher relative mRNA levels than vehicle-treated rats (*p* < 0.001) and those treated with 5 mg/kg (*p* < 0.001).

##### 3.2.1.4. Cx3cr1 at 240 min.

The analysis revealed significant differences in *Cx3cr1* mRNA expression between male and female rats under basal conditions (t_6_ = 2.44; *p* = 0.045), with females showing significantly lower relative mRNA levels compared with vehicle-treated males (Fig. 3J).

In male rats, there was a significant main effect of “dose of cocaine” on *Cx3cr1* mRNA expression at 240 min (F_3,21_ = 5.56; *p* = 0.006) (Fig. 3K). *Post hoc* tests showed that males treated with 15 mg/kg of cocaine had higher relative mRNA levels than vehicle-treated rats (*p* < 0.01) and rats treated with 5 mg/kg (*p* < 0.05). The analysis also revealed a significant main effect of “dose of cocaine” at 240 min in female rats (F_3,20_ = 29.20; *p* < 0.001) (Fig. 3L). *Post hoc* tests showed that females treated with 15 and 30 mg/kg displayed higher relative mRNA levels than vehicle-treated rats (*p* <0.001) and those treated with 5 mg/kg (*p* < 0.001).

#### 3.2.2. Repeated cocaine treatment

##### 3.2.2.1. Cx3cl1.

**Fig. 4.**
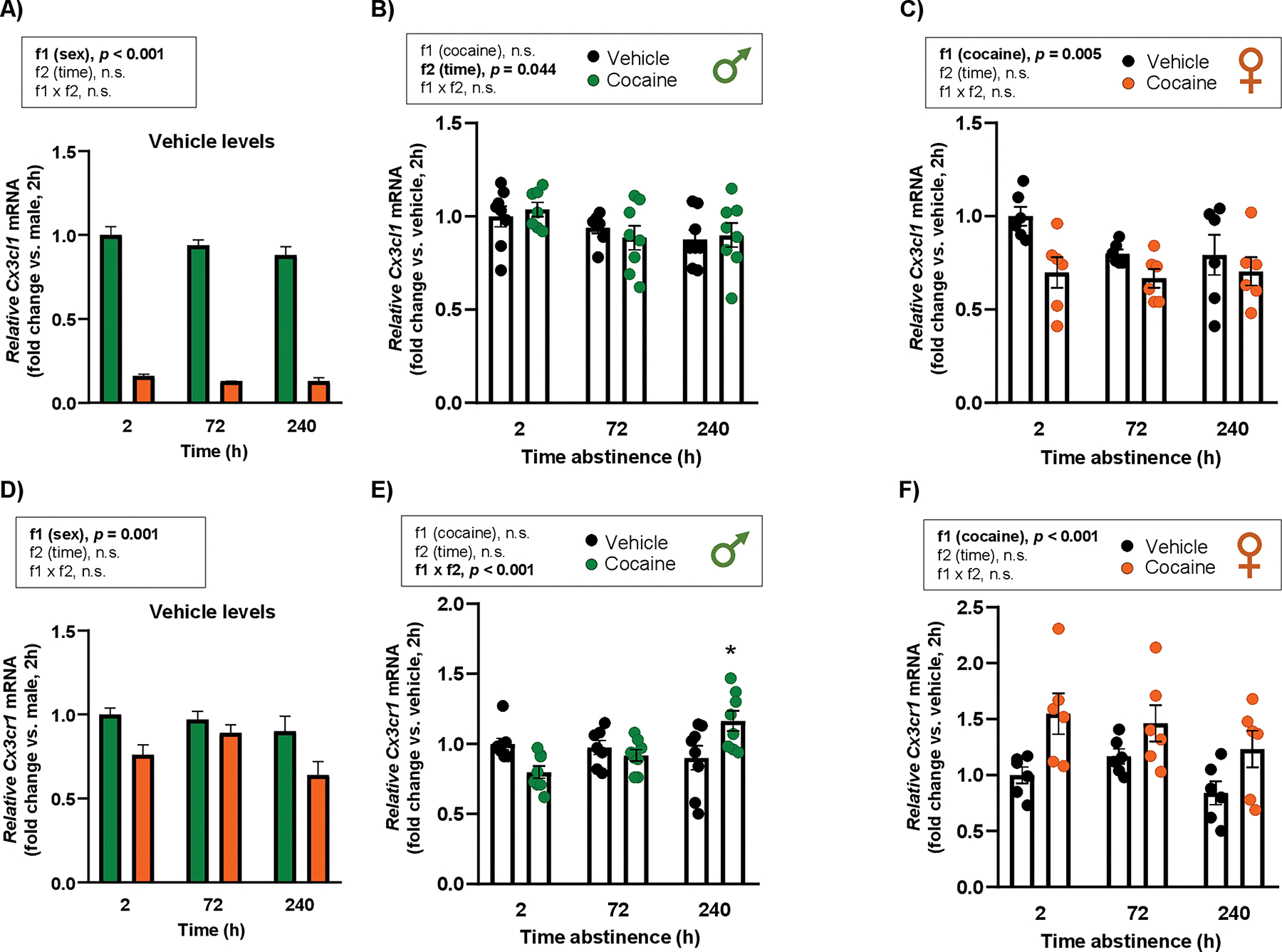
Effects of repeated cocaine exposure on the relative mRNA expression of *Cx3cl1* and *Cx3cr1* in the hippocampus of rats. Basal *Cx3cl1* mRNA levels in male and female control rats at different time points after vehicle administration (fold change vs. males, dose 0) **(A)**. Relative *Cx3cl1* mRNA levels in male **(B)** and female rats **(C)** 2, 72, and 240 h after vehicle or cocaine treatment. Basal *Cx3cr1* mRNA levels in male and female control rats at different time points after vehicle administration (fold change vs. males, dose 0) **(D)**. Relative *Cx3cr1* mRNA levels in male **(E)** and female rats **(F)** 2, 72, and 240 h after vehicle or cocaine treatment. Relative mRNA levels were analyzed by two-way ANOVA. Bars represent the mean ± SEM (6–8 rats/group). (*) *p* < 0.05 indicates significant differences compared with their respective vehicle group.

First, vehicle-treated rats were compared between males and females. A two-way ANOVA revealed a significant main effect of “sex” (F_1,34_ = 722.1; *p* < 0.001) (Fig. 4A). Male rats treated with vehicle had higher relative *Cx3cl1* mRNA levels in the hippocampus than females at all time points.

In male rats, a two-way ANOVA indicated a significant main effect of “time of abstinence” (F_2,40_ = 3.38; *p* = 0.044), with no main effect of cocaine exposure or interaction (Fig. 4B). In female rats, a two-way ANOVA showed a significant main effect of “cocaine” (F_1,30_ = 9.27; *p* = 0.005), with no main effect of “time of abstinence” or interaction (Fig. 4C). Thus, female rats treated with cocaine displayed lower relative *Cx3cl1* mRNA levels than vehicle-treated rats.

##### 3.2.2.2. Cx3cr1.

When comparing vehicle-treated males and females, the statistical analysis revealed a significant main effect of “sex” (F_1,33_ = 12.89; *p* = 0.001) on *Cx3cr1* mRNA expression (Fig. 4D). Male rats treated with vehicle had higher relative *Cx3cr1* mRNA levels than female rats.

In male rats, there was a significant interaction between “cocaine” and “time of abstinence” (F_2,41_ = 8.34; *p* <0.001) (Fig. 4E). *Post hoc* tests showed that males treated with cocaine exhibited higher relative *Cx3cr1* mRNA levels than vehicle-treated rats at 240 h (*p* < 0.05). In female rats, a two-way ANOVA revealed a significant main effect of “cocaine” (F_1,30_ = 14.16; *p* < 0.001), but no main effect of “time of abstinence” or interaction (Fig. 4F). Accordingly, cocaine-treated females showed higher relative *Cx3cr1* mRNA levels than vehicle-treated rats.

### 3.3. Effects of cocaine exposure on CX_3_CL1 concentrations in the plasma of rats

#### 3.3.1. Acute cocaine treatment

##### 3.3.1.1. CX_3_CL1 at 30 min.

**Fig. 5.**
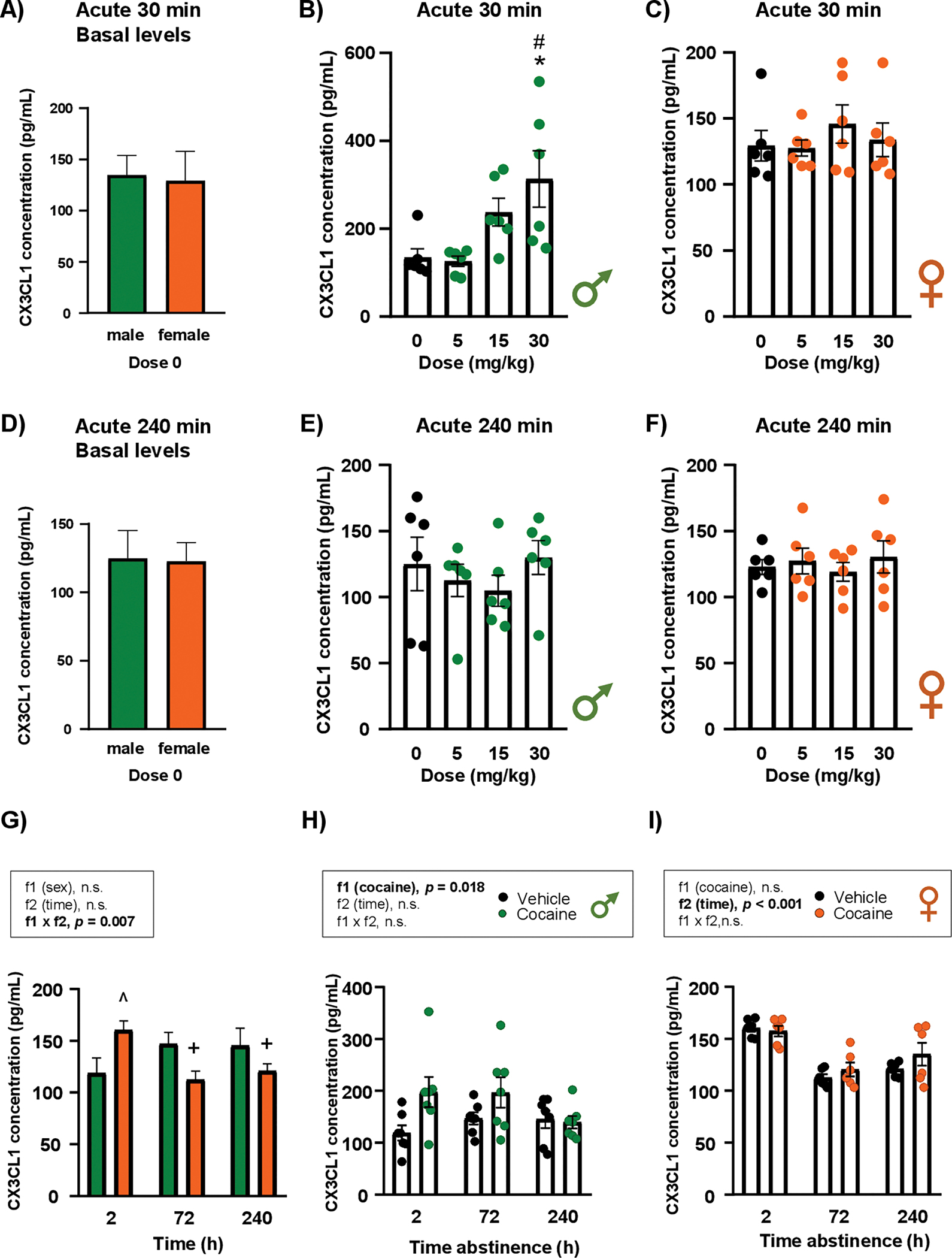
Effects of acute and repeated cocaine exposure on plasma CX_3_CL1 concentrations in rats. Basal CX_3_CL1 concentrations in male and female control rats 30 min after vehicle administration **(A)**. Plasma CX_3_CL1 concentrations in male **(B)** and female rats **(C)** 30 min after vehicle or cocaine treatment. Basal CX_3_CL1 concentrations in male and female control rats 240 min after vehicle administration **(D)**. Plasma CX_3_CL1 concentrations in male **(E)** and female rats **(F)** 240 min after vehicle or cocaine treatment. Basal CX_3_CL1 concentrations in male and female control rats at different time points after vehicle administration **(G)**. Plasma CX_3_CL1 concentrations in male **(H)** and female rats **(I)** 2, 72, and 240 h after vehicle or cocaine treatment. Basal CX_3_CL1 concentrations were analyzed using Student’s *t*-test (Fig. 5A and 5D) or by two-way ANOVA (Fig. 5G). CX_3_CL1 concentrations from acute treatment in male and female rats were analyzed by one-way ANOVA (.Fig. 5B, 5C, 5E, and 5F). CX_3_CL1 concentrations from repeated treatment in male and female rats were analyzed by two-way ANOVA (Fig. 5H and 5I). Bars represent the mean ± SEM (6–8 rats/group). (^) *p* < 0.05 indicates significant differences compared with control males. (+) *p* < 0.05 indicates significant differences compared with control females at 2 h. (*) *p* < 0.05 indicates significant differences compared with the vehicle group and (#) *p* < 0.05 indicates significant differences compared with the group treated with 5 mg/kg.

There were no differences in CX_3_CL1 concentrations between male and female rats under basal conditions (vehicle or dose 0) (Fig. 5A).

In male rats, a one-way ANOVA showed a significant main effect of “dose of cocaine” on plasma CX_3_CL1 concentrations at 30 min (F_3,21_ = 4.39; *p* = 0.016) (Fig. 5B). *Post hoc* comparisons indicated that males treated with 30 mg/kg had significantly higher CX_3_CL1 concentrations than vehicle-treated rats (*p* < 0.05) and those treated with 5 mg/kg (*p* < 0.05). In female rats, no significant main effect of “dose of cocaine” was found for CX_3_CL1 concentrations at 30 min (Fig. 5C).

##### 3.3.1.2. CX_3_CL1 at 240 min.

As at 30 min, no differences in basal CX_3_CL1 concentrations were observed between male and female vehicle-treated rats (Fig. 5D).

Notably, one-way ANOVAs in both sexes showed no significant main effects of “dose of cocaine” on plasma CX_3_CL1 concentrations at 240 min (Fig. 5E and F).

#### 3.3.2. Repeated cocaine treatment

CX_3_CL1 concentrations were analyzed for all rats by two-way ANOVA following repeated cocaine treatment at three different abstinence times. No significant main effects appeared when comparing males and females; however, there was a significant interaction between “sex” and “time” (F_2,33_ = 5.78; *p* = 0.007) (Fig. 5G). Specifically, among vehicle-treated rats, females exhibited higher plasma CX_3_CL1 concentrations than males at 2 h of abstinence (*p* < 0.05). At 72 and 240 h, however, females showed significantly lower CX_3_CL1 concentrations compared with those at 2 h, with no differences relative to males at these later time points.

Plasma CX_3_CL1 concentrations were then analyzed separately in male and female rats, comparing cocaine- and vehicle-treated rats at each abstinence time. In male rats, the analysis revealed a significant main effect of “cocaine” (F_1,36_ = 6.11; *p* = 0.018), with cocaine-treated rats displaying higher CX_3_CL1 concentrations than vehicle-treated rats, particularly at 2 and 72 h (Fig. 5H). In contrast, the analysis in female rats showed a significant main effect of “time” on CX_3_CL1 concentrations (F_2,30_ = 25.78; *p* < 0.001), with lower concentrations at 72 and 240 h compared with 2 h (Fig. 5I).

### 3.4. CX_3_CL1 concentrations in the plasma of patients with CUD

A cohort of patients with CUD and healthy controls was included in the study to assess plasma CX_3_CL1 concentrations. Patients with a lifetime diagnosis of CUD (*N* = 88) were recruited from an outpatient treatment program for cocaine use during abstinence (Table S2). Control participants (*N* = 30) with no history of substance use disorders or significant psychiatric conditions were recruited from a pool of volunteers.

#### 3.4.1. Sample characteristics

Patients with CUD had a median age of 34.0 years, median body mass index (BMI) of 25.5 kg/m^2^, and a higher proportion of males (*N* = 69) compared with females (*N* = 19). Psychiatric comorbidity was common (36.4 %), predominantly mood (33 %) and personality (30 %) disorders. Although cocaine was the primary substance motivating outpatient treatment, additional substance use disorders were identified with PRISM in 27 % of patients, most frequently alcohol use disorder (24 %). Regarding CUD, patients exhibited a median abstinence duration of 75 days and a median CUD severity score of 9 out of 11 criteria.

Control participants were balanced in age, BMI, and sex ratio (24 males and 6 females; 20 % females).

#### 3.4.2. Plasma CX_3_CL1 concentrations and abstinence

We investigated the influence of abstinence duration on plasma CX_3_CL1 concentrations in patients with CUD, following the characterization conducted in the animal models.

**Fig. 6.**
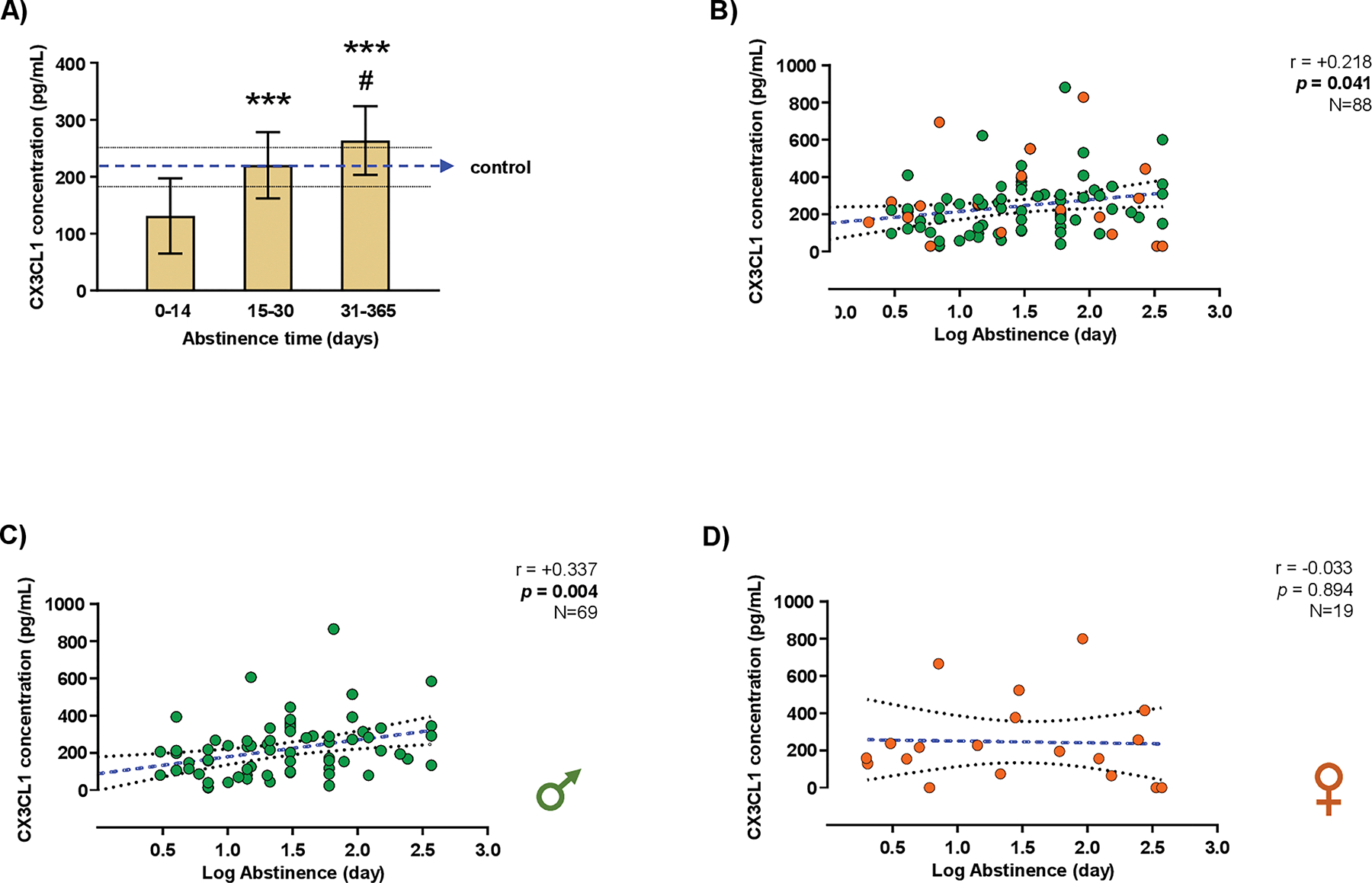
Plasma CX_3_CL1 concentrations in patients with CUD. Plasma CX_3_CL1 concentrations in CUD patients grouped by the duration of cocaine abstinence **(A)**. Correlation analysis between plasma CX_3_CL1 concentrations and the duration of cocaine abstinence (days) in all CUD patients **(B)**, men with CUD **(C)**, and women with CUD **(D)**. Data were analyzed using one-way ANOVA (Fig. 6A). Bars represent the mean ± 95 % confidence interval (CI) [control: N = 30 (24 males and 6 females); 0–14 days: N = 28 (7 females and 21 males); 15–30 days: N = 22 (4 females and 18 males); and 31–365 days: N = 38 (8 females and 30 males)]. (***) *p* < 0.001 indicates significant differences compared with the 0–14 day subgroup, (#) *p* < 0.05 indicates significant differences compared with the 15–30 day subgroup. The blue arrow and the black dashed lines denote the mean and 95 % CI of CX_3_CL1 concentrations in the control group. Correlations were assessed using Pearson’s correlation coefficient (*r*) between log_10_-transformed days of cocaine abstinence and plasma CX_3_CL1 concentrations (Fig. 6B, 6C, and 6D). Each dot represents an individual value; the blue line indicates the linear fit, and the black dashed lines indicate the 95 % CI.

Initially, CX_3_CL1 concentrations were measured in both control participants and patients with CUD. No significant between-group differences were observed (CUD: 226.4 pg/mL, 95 %CI = 186.9–265.9; controls: 218.2 pg/mL, 95 %CI = 180.1–256.2). Patients with CUD were then stratified into three subgroups based on their duration of cocaine abstinence [0–14 days (25 % females), 15–30 days (18 % females), and 31–365 days (21 % females); Fig. 6A). A one-way ANOVA revealed a significant main effect of abstinence duration on CX_3_CL1 concentrations (F_2,85_ = 35.03; *p* < 0.001), with a progressive increase across subgroups. At 31–365 days of abstinence, CX_3_CL1 concentrations matched those of the control group and were significantly higher than those observed in the 0–14 day subgroup (*p* < 0.05).

Furthermore, a significant positive correlation was identified between plasma CX_3_CL1 concentrations and the duration of cocaine abstinence (log_10_-transformed values) (*r* = +0.22; *p* < 0.05) (Fig. 6B). When analyzing male and female patients separately, a significant correlation between plasma CX_3_CL1 concentrations and abstinence duration was observed in men (*r* = +0.34; *p* < 0.01) (Fig. 6C), but not in women (Fig. 6D).

## 4. Discussion

This study demonstrates that both acute and repeated exposure to cocaine modulates the mRNA expression of *Cx3cl1* and *Cx3cr1* in the amygdala and hippocampus, revealing pronounced sex-dependent patterns. Female control rats consistently exhibited lower baseline mRNA levels of both genes, while acute cocaine produced dose- and time-dependent alterations, with females showing heightened sensitivity. Repeated exposure further modulated mRNA expression depending on sex, brain region, and abstinence duration, indicating dynamic regulation of the CX_3_CL1/CX_3_CR1 axis throughout the course of chronic cocaine use. It is important to note that these findings are limited to transcriptional regulation; no direct protein quantification was performed, and any functional implications should therefore be considered hypothetical. These central effects were paralleled by changes in plasma CX_3_CL1 concentrations, which may reflect, but do not confirm, peripheral manifestations of neuroimmune adaptations.

The region-specific patterns observed in the amygdala and hippocampus align with their distinct functional roles in addiction-related neurobiology. The involvement of the amygdala in emotional regulation and conditioned cue reactivity may explain its rapid transcriptional responses to acute cocaine, while the hippocampus, critical for contextual memory and stress regulation, showed more sustained transcriptional changes during repeated exposure and abstinence. These patterns are consistent with human imaging evidence indicating dopaminergic activation of both regions in response to cocaine cues (Fotros et al., 2013) and with preclinical findings implicating the dorsal hippocampus in relapse behavior after prolonged abstinence (Werner et al., 2020). Additionally, microglia-focused work indicates that limbic circuits, including amygdala and hippocampus, undergo stress-related microglial remodeling and cytokine regulation (Nowak et al., 2025), which could be consistent with the sex-dependent transcriptional changes observed in our rat model. Notably, the hippocampal *Cx3cl1* elevations detected shortly after acute cocaine exposure resemble the transient increases in hippocampal CX_3_CL1 protein reported previously in mice following repeated cocaine administration (Montesinos et al., 2020), supporting the idea that this chemokine is rapidly, but not persistently, regulated by cocaine across paradigms. Together, these data support the idea that cocaine modulates the CX_3_CL1/CX_3_CR1 axis within brain circuits critical for integrating emotional, cognitive, and stress-related processes, although confirmation at the protein and functional levels is still needed.

The consistently lower basal *Cx3cl1* and *Cx3cr1* mRNA expression in female rats across both brain regions may be attributed to the influence of sex hormones on microglial function. Specifically, estrogen has been shown to modulate neuroimmune signaling, including CX_3_CL1-mediated microglial activity, which is essential for maintaining neuronal homeostasis (Baker et al., 2004; Mor et al., 1999; Wu et al., 2016). Given the role of the CX_3_CL1/CX_3_CR1 axis in microglia-neuron communication, neuroinflammation, and synaptic plasticity, these hormonal influences might contribute to the observed sex-specific transcriptional patterns and could partly explain differences in vulnerability to neuropsychiatric conditions (Luo et al., 2019; Pawelec et al., 2020).

Acute cocaine induced region- and sex-specific transcriptional responses. In the amygdala, male rats exhibited a biphasic *Cx3cl1* response at 30 min, with suppression at low doses and elevation at high doses, possibly reflecting a compensatory neuroimmune mechanism in response to cocaine-induced stress (Walter and Crews, 2017). This effect was transient and reversed at 240 min, suggesting a progressive shift toward proinflammatory signaling. In contrast, females exhibited a dose-dependent upregulation of both *Cx3cl1* and *Cx3cr1* mRNA in the amygdala, potentially reflecting estrogen-mediated sensitization of the CX_3_CL1 pathway (Hou et al., 2016). In the hippocampus, acute cocaine induced transient increases in *Cx3cl1* expression in both sexes at 30 min, similar in direction to the hippocampal CX_3_CL1 protein increases observed by Montesinos et al. in mice after repeated exposure (Montesinos et al., 2020). By 240 min, these effects had dissipated. Females showed a more pronounced and sustained increase in *Cx3cr1* expression across doses and time points, although confirmation at the protein level will be essential; this pattern is consistent with previous reports of stronger inflammatory responses in females following substance exposure, including upregulation of microglia-associated genes and chemokines (Barton et al., 2017; Pascual et al., 2017).

Repeated cocaine exposure further emphasized the sex-specific modulation of the CX_3_CL1/CX_3_CR1 axis. In the amygdala, vehicle-treated animals showed a transient increase in *Cx3cl1* expression, peaking at 72 h, likely due to circadian influences or low-grade inflammatory responses to repeated handling (Corsi et al., 2022; Kim et al., 2024). Cocaine exposure, however, selectively reduced *Cx3cl1* in females during abstinence, which may reflect an adaptive suppression of neuroinflammation, as cocaine has been shown to induce microglial overactivation and IL-1β release through CX_3_CL1-linked mechanisms (Montesinos et al., 2020; Pe’er-Nissan et al., 2024). Males showed an early increase in *Cx3cr1* mRNA, while females exhibited delayed upregulation, potentially as a compensatory transcriptional response to diminished *Cx3cl1* expression.

In the hippocampus, baseline sex differences persisted, with female rats showing significantly lower expression of both genes; however, repeated cocaine produced more nuanced effects. While *Cx3cl1* expression remained stable in males, it was significantly reduced in females. In contrast, *Cx3cr1* was upregulated in both sexes, but more prominently and persistently in females. These results may suggest a prolonged transcriptional signature of microglial activation, which could be consistent with increased susceptibility to neuroinflammation and related behavioral consequences, such as cognitive dysfunction or emotional dysregulation, during abstinence. Supporting this, Medina-Vera et al. (2025) reported that CX_3_CR1-deficient mice exposed to adolescent stress and alcohol exhibited dysfunctional neuroinflammatory responses and maladaptive coping behaviors (Medina-Vera et al., 2025), underscoring the potential role of CX_3_CR1 in regulating stress resilience and neuroimmune responses in the context of substance use, although our data cannot directly confirm such functional outcomes.

Peripheral CX_3_CL1 concentrations reflected some of the central transcriptional patterns. Following acute cocaine exposure, elevated plasma CX_3_CL1 concentrations were observed only in males receiving high-dose cocaine, whereas repeated cocaine exposure led to sustained increases in males during early abstinence. In contrast, females did not exhibit significant changes in plasma CX_3_CL1, aligning with a distinct regulatory mechanism, potentially influenced by anti-inflammatory properties of estrogens (Fox et al., 2008; Peart et al., 2022). Notably, repeated vehicle injections alone induced sex-dependent alterations in plasma CX_3_CL1, suggesting that even mild stressors may differentially engage neuroimmune pathways in males and females (Deak et al., 2015; Sood et al., 2022).

The translational relevance of these findings is underscored by our human data. Although no significant differences in plasma CX_3_CL1 concentrations were detected between patients with CUD and healthy controls, consistent with earlier studies (Araos et al., 2015; Pedraz et al., 2015), stratification by abstinence duration revealed a significant increase in CX_3_CL1 concentrations over time. This time-dependent rise parallels observations by Pedraz et al. that immune alterations in CUD may be more evident with sustained abstinence than during active use (Pedraz et al., 2015), and aligns with Araos et al., who in abstinent patients reported associations between CX_3_CL1 and addiction severity (Araos et al., 2015); in a parallel rodent experiment, the same study detected cocaine-dependent changes in circulating cytokines/chemokines, supporting the peripheral sensitivity of these markers to cocaine exposure. A moderate positive correlation was observed in the full sample, with a notably stronger association in men, which parallels the sex-specific patterns seen in our preclinical data. This interpretation should also consider clinical heterogeneity: patients with CUD showed high psychiatric comorbidity and a substantial proportion with additional substance use disorders (predominantly alcohol), both of which could influence plasma CX_3_CL1. Although we did not stratify analyses by these factors, the positive correlation with abstinence remained evident in men. In women, the lack of significance may reflect limited power (*N* = 19), potential sex differences in basal CX_3_CL1 levels, and sex-specific comorbidity profiles. While these findings do not establish causality, they are compatible with the hypothesis that this chemokine might serve as a sex-sensitive biomarker (or as part of a composite biosignature, together with other biomolecules) and potential therapeutic target for cocaine addiction.

In conclusion, these findings provide compelling evidence that cocaine exposure modulates the transcriptional expression of the CX_3_CL1/CX_3_CR1 axis in a dose-, time-, and sex-dependent manner across key brain regions implicated in addiction. Because the present data are limited to mRNA measures, these interpretations should be considered as hypotheses requiring confirmation at the protein and functional levels. Nevertheless, the parallel patterns observed in plasma CX_3_CL1 concentrations in both animals and humans suggest a potential peripheral reflection of central transcriptional changes. The consistent sex differences observed in both preclinical and clinical contexts highlight the importance of integrating sex-based analyses into addiction research. Future efforts should aim to determine whether these transcriptional signatures translate into protein-level changes and functional outcomes relevant to addiction vulnerability, withdrawal severity, and relapse risk.

## Supplementary Material

Table S1

Table S2

## Data Availability

Individual animal-level data are fully available in the article. Due to ethical and legal restrictions related to patient confidentiality, human data supporting the findings of this study are available upon reasonable request from the corresponding author(s).

## Appendix A. Supplementary data

Supplementary data to this article can be found online at https://doi.org/10.1016/j.pnpbp.2025.111482.
