# Supplementary material for: Sex-specific dysregulation of the CX3CL1/CX3CR1 Axis following cocaine exposure: Translational evidence for a potential biomarker of abstinence
Source: Prog Neuropsychopharmacol Biol Psychiatry. Author manuscript; Available in PMC 2026 Feb 2. (PMC12862095; doi:10.1016/j.pnpbp.2025.111482)
Supplement: Table S1 [file NIHMS2134873-supplement-Table_S1.docx]

**Table S1.** TaqMan® Gene Expression Assays used for RT-qPCR analysis in rat brain samples.

| Gene | Description | Assay ID | RefSeq Accession No. | Amplicon Length |
| --- | --- | --- | --- | --- |
| *Cx3cl1* | Chemokine (C-X3-C motif) ligand 1 | Rn00667869_m1 | NM_031144.3 | 91 |
| *Cx3cr1* | Chemokine (C-X3-C motif) receptor 1 | Rn00593186_m1 | NM_134455.1 | 90 |
| *Actb* | Beta-actin (housekeeping gene) | Rn02134446_s1 | NM_133534.1 | 124 |

All assays were obtained from Thermo Fisher Scientific (Waltham, MA, USA)
