# Supplementary material for: Sex-specific dysregulation of the CX3CL1/CX3CR1 Axis following cocaine exposure: Translational evidence for a potential biomarker of abstinence
Source: Prog Neuropsychopharmacol Biol Psychiatry. Author manuscript; Available in PMC 2026 Feb 2. (PMC12862095; doi:10.1016/j.pnpbp.2025.111482)
Supplement: Table S2 [file NIHMS2134873-supplement-Table_S2.docx]

**Table S2.** Clinical characteristics of patients with CUD.

| **Variable** | | **Patients with CUD**  **N=88** |
| --- | --- | --- |
| **Sex**  **[N (%)]** | Male  Female | 69 (78.4)  19 (21.6) |
| **Age, years**  **(median and IQR)** | | 34.0 (29.0-39.0) |
| **BMI, kg/m^2^**  **(median and IQR)** | | 25.5 ( 22.3-28.6) |
| **Comorbid substance use disorders**  **[N (%)]** | Yes  No | 24 (27.3)  64 (72.7) |
|  | Alcohol  Cannabis  Others | 21 (23.9)  6 (6.8)  4 (4.5) |
| **Comorbid mental disorders**  **[N (%)]** | Yes  No | 32 (36.4)  56 (63.6) |
|  | Mood disorders  Anxiety disorders  Psychotic disorders  Personality disorders | 29 (33.0)  22 (25.0)  12 (15.9)  26 (29.5) |
| **Smoking**  **[N (%)]** | Yes  No  Ex-smoker | 51 (58.0)  29 (33.0)  8 (9.1) |
| **Cocaine abstinence, day**  **(median and IQR)** | | 75.0 (15.0-115.5) |
| **CUD severity score (0-11)**  **(median and IQR)** | | 9.0 (8.0-10.0) |

Abbreviations: BMI = body mass index; CUD = cocaine use disorder; IQR = interquartile range.
